# Assessing the Biodegradability of PHB-Based Materials with Different Surface Areas: A Comparative Study on Soil Exposure of Films and Electrospun Materials

**DOI:** 10.3390/polym15092042

**Published:** 2023-04-25

**Authors:** Kristina G. Gasparyan, Polina M. Tyubaeva, Ivetta A. Varyan, Alexandre A. Vetcher, Anatoly A. Popov

**Affiliations:** 1 Department of Physical Chemistry of Synthetic and Natural Polymer Compositions, Emanuel Institute of Biochemical Physics, Russian Academy of Sciences, 4 Kosygina Street, 119334 Moscow, Russiaanatoly.popov@mail.ru (A.A.P.); 2 Academic Department of Innovational Materials and Technologies Chemistry, Plekhanov Russian University of Economics, 36 Stremyanny Per., 117997 Moscow, Russia; 3 Complementary and Integrative Health Clinic of Dr. Shishonin, 5 Yasnogorskaya Str., 117588 Moscow, Russia; 4 Institute of Biochemical Technology and Nanotechnology, Peoples’ Friendship University of Russia (RUDN), 6 Miklukho-Maklaya St., 117198 Moscow, Russia

**Keywords:** biodegradable polymers, poly-3-hydroxybutyrate, electrospinning, fiber, film, decomposition, biodegradation in soil

## Abstract

Due to the current environmental situation, biopolymers are replacing the usual synthetic polymers, and special attention is being paid to poly-3-hydroxybutyrate (PHB), which is a biodegradable polymer of natural origin. In this paper, the rate of biodegradation of films and fibers based on PHB was compared. The influence of exposure to soil on the structure and properties of materials was evaluated using methods of mechanical analysis, the DSC method and FTIR spectroscopy. The results showed rapid decomposition of the fibrous material and also showed how the surface of the material affects the rate of biodegradation and the mechanical properties of the material. It was found that maximum strength decreased by 91% in the fibrous material and by 49% in the film. Additionally, the DSC method showed that the crystallinity of the fiber after exposure to the soil decreased. It was established that the rate of degradation is influenced by different factors, including the surface area of the material and its susceptibility to soil microorganisms. The results obtained are of great importance for planning the structure of features in the manufacture of biopolymer consumer products in areas such as medicine, packaging, filters, protective layers and coatings, etc. Therefore, an understanding of the biodegradation mechanisms of PHB could lead to the development of effective medical devices, packaging materials and different objects with a short working lifespan.

## 1. Introduction

In recent years, people have become more aware of environmental pollution and in this regard have begun to actively develop the study and application of bioplastics as an excellent alternative to disposable products which can be produced and decomposed naturally by microorganisms [1]. Currently, many bioplastics are used, including polylactide (PLA), polycaprolactone (PCL) and poly-3-hydroxybutyrate (PHB), which can replace the usual petroleum-based plastics [2].

Of special interest is a natural biodegradable polyester PHB which is synthesized from renewable natural sources. This polymer has good service properties (for example, high thermal stability over a broad temperature interval up to 150 °C), is biodegradable, biocompatible with the human body and can be sterilized by various physical and chemical methods [3,4]. Moreover, PHB can serve as a perfect polymer host matrix for the development of efficient materials for different areas including medical devices, packaging materials and different objects with a short working lifespan. However, its main advantage is considered to be its good biodegradability, whereby the problem of further utilization of PHB-based materials can be easily solved [5,6].

**Figure 1:**
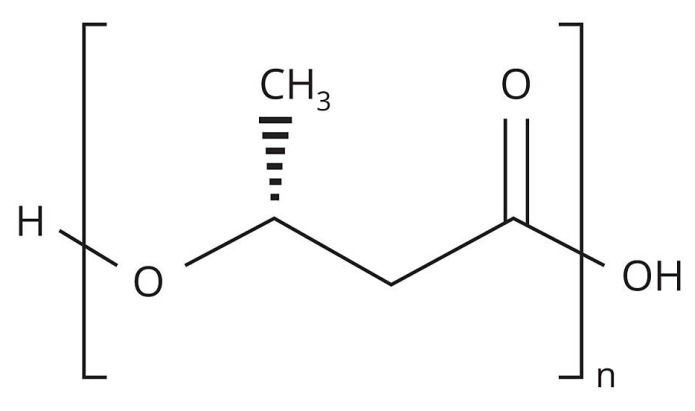
Structural formula of PHB.

PHB (Figure 1) is the first bioplastic and was discovered in 1926 by the French researcher Maurice Lamoine. His discovery was ignored for many decades because coal and oil were inexpensive, but in the mid-1970s the world economy experienced an oil crisis and began to explore alternatives. In nature, this polymer is synthesized by some types of microorganisms that belong to the genres *Alcaligenes*, *Azobacter*, *Bacillus* and *Pseudomonas* [3], it is found in the cytoplasm of microbial cells in the form of granules and usually makes up more than 40% of their dry weight [4].

So far, PHB is not particularly common because of its fragility: it does not stretch well and breaks easily compared to other types of bioplastics [5,6]. To improve its mechanical and other desired properties, various substances are added: polymers, nanoparticles, metals, plasticizers and the other additives given in Table 1. PHB is used in many fields: the textile industry, fabric engineering, biomedical products, food industry (disposable trays, cups, plates) [7,8]. Moreover, it is of great importance that the modification of PHB does not affect its high biodegradability [9].

**Figure 2:**
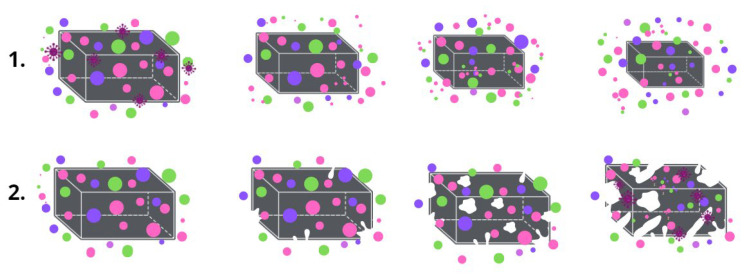
The mechanism of biodegradation: (**1**) surface, (**2**) bulk.

Despite the popularity of PHB, its biodegradation mechanisms are still an area of a great interest [15]. The hydrolytic and enzymatic degradation of PHB have been well studied separately [16,17]. The essence of the PHB biodegradation mechanism is the activity of the depolymerase enzymes, which provide three key stages of the decomposition process: transition from a high-molecular compound to monomers and oligomers; transition from monomers and oligomers to biomass; transition from biomass to CO_2_ and H_2_O [18]. It is the enzymes that catalyze the hydrolytic erosion of the surface that triggers the process of biodegradation [19,20]. Of course, the biodegradation of the material proceeds as a complex process that is influenced by many factors, especially: surface roughness, available surface area, hydrophobicity, charge, chemical and supramolecular structure, etc. [21]. However, during composting in controlled conditions the process of biodegradation will be largely determined by the specifics of the structural organization of the material. Two key ways in which material are destroyed should be distinguished (Figure 2): surface biodegradation and bulk biodegradation. In the main part, porous material with a highly developed surface area undergoes bulk destruction which is accompanied by autocatalytic hydrolysis, and dense monolithic bodies such as films undergo surface destruction [22].

Considering that over the past 5 years PHB production has increased four-fold [23], this study of PHB biodegradation was carried out in various environments, such as: soil, drinking water, the marine environment, etc. [8,24]. In the main part, in the studies conducted the loss of mass of the composite based on PHB was observed. Among the key environmental factors determining the rate of biodegradation, the following were particularly noted: type of microorganism, temperature, pH, humidity of the medium and availability of oxygen and nutrients in the medium [25]. Furthermore, it was found that degradation proceeded faster in an alkaline medium [26] and at a higher temperature [27]. The mechanism of the PHB decomposition in the soil is almost aerobic, accompanied by hydrolysis and oxidation by soil components. Additionally, it is known that the most active microorganisms consume polymers of monocarboxylic acids, with polyhydroxyalkanoates occupy a leading position among these polymers [28]. At the moment, different groups of bacteria and fungi are particularly capable of destroying PHB molecular chains: gram-positive and gram-negative bacteria of the genus *Streptomyces*, *classes of Firmicutes*, *Pseudomonas lemoigne*, *Comamonas* sp., *Acidovorax faecalis*, *Aspergillus fumigatus* and *Variovorax paradoxus* [28].

Considering the designated issues, the main purpose of this study was to find out how the surface area of PHB materials affects the rate of the biodegradation process. Electrospun fibrous material was chosen as the model of the highly developed porous material and a film was chosen as the comparison object.

This work offers a simple and versatile approach to studying, under natural conditions, the surface area of PHB-based nonwoven fibrous materials in comparison to film materials. This comprehensive approach may provide a more thorough understanding of the effects exposure to soil has on the structure and properties of the biomaterials for different properties, since this article compares, for the first time, the factors characterizing the contribution made by the surface to the speed and type of the mechanism of initiation of the biodegradation process.

## 2. Materials and Methods

### 2.1. Materials

A biopolymer of natural origin—poly-3-hydroxybutyrate (PHB)—was used in this work. Commercial PHB was obtained by microbial synthesis (16F series, production by BIOMER, Frankfurt, Germany), and was characterized by 60% of crystalline phase, 206 kDa of molecular weight, 1.248 g/cm^3^ of density, melt flow index (MFI) = 10 g/10 min (180 °C, 5 kg).

Materials were obtained from PHB using two methods: electrospinning and pressing.

PHB films were obtained by pressing on the laboratory hydraulic press (Moscow, Russia). The advantages of the pressing method are the simple design of the molds and the relatively low cost of the equipment [29]. This method made it possible to obtain uniform thin films comparable to nonwoven analogues. The dosage of the PHB for pressing was 1.1 g, the temperature on the heating plates was 180–190 °C and pressure was 50–60 MPa. The duration of pressing was 2 min. The obtained samples of PHB films were cooled in water at a temperature of about 20 °C for 5 min. The thickness of the PHB films was 130 ± 0.098 µm.

**Figure 3:**
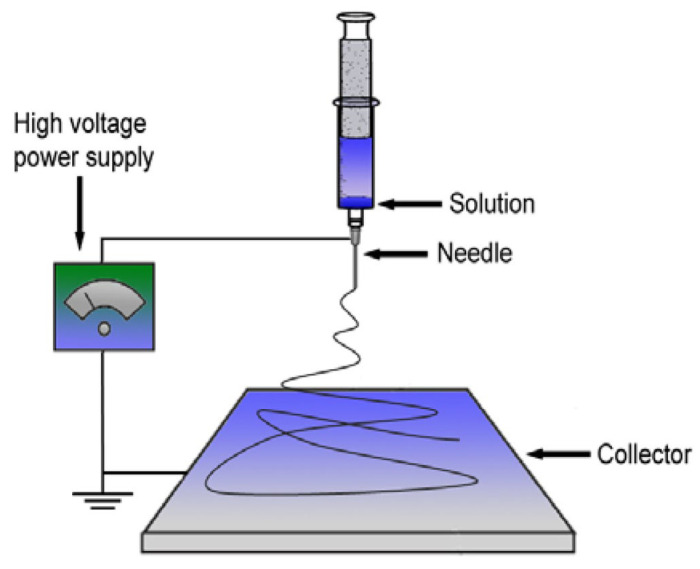
The scheme of the single-capillary laboratory unit for electrospinning [31].

PHB fibers were obtained by using the electrospinning [30] method on the single-capillary laboratory unit EFV-1 (Moscow, Russia). The scheme and the main structural components, including the solution reservoir, a stainless-steel needle, a high-voltage power supply and a vertically grounded collector, are shown in Figure 3 [31]. A homogeneous polymer solution was prepared by dissolving PHB in chloroform at 7% (*w*/*v*). The electrical conductivity of the solution was 10 μS/cm and its viscosity was 1.0 Pa s. The layers of the fibrous material were obtained from 25 mL of PHB solution. The conditions of the electrospinning process were voltage 17 kV, the distance between the electrodes—200 mm and the gas pressure on the solution—14 kg (f) cm^−2^. The electrospun materials were dried at 24 °C for 48 h to remove residual solvents and moisture [32]. The thickness of the PHB fibrous materials was 130 ± 0.047 µm.

### 2.2. Methods

The main methods used in this work to study the structure and properties of materials in the process of biodegradation were scanning electron and optical microscopy, determination of morphology characteristics (surface density, thickness and porosity), mechanical analysis, differential scanning calorimetry, infrared spectroscopy and indoor exposure by composting in the soil.

#### 2.2.1. Scanning Electron Microscopy (SEM)

The determination of the morphology of the surface area of the PHB materials was carried out by SEM observations using a Tescan VEGA3 microscope (Wurttemberg, Czech Republic). The SEM microphotographs were obtained at an accelerating voltage of 20 kV. Before scanning, the samples were vacuumized and covered with a layer of patina.

#### 2.2.2. Optical Microscopy

The determination of structural changes, such as the diameter of fibers, changes in morphology etc., were carried out using an Olympus BX43 (Olympus, Japan, Tokyo). The main morphological properties of the fibers were measured by the micrograph using Olympus Stream Basic software (Tokyo, Japan).

#### 2.2.3. Surface Density, Thickness and Porosity Estimation

The determination of the surface density was carried out using the analytical weighing-machine Balance XPR106DUHQ/A (Mettler Toledo, Columbus, OH, USA). Surface density, δ, g/cm^3^, was calculated as:

(1) $$\delta =\frac{m}{l\times B\times b}$$

where *m* is the weight of the sample; *l* is the length; *B* is the width; *b* is the thickness. The average value was obtained from 10 measurements taken at different parts of the sample.

The determination of the porosity was carried out using the analytical weighing-machine Balance XPR106DUHQ/A (Mettler Toledo, Columbus, OH, USA). Porosity, W, %, was calculated as:

(2) $$W=\left(1-\frac{V_{PHB}}{V_{s}}\right)\times 100\%$$

where $V_{f}$ is the volume of the polymer; $V_{s}$ is the volume of the the sample of the material. The average value was obtained from 10 measurements taken at different parts of the sample.

The determination of the thickness was carried out using the analytical weighing-machine Digital micrometer 0–25 mm, NMD-165D (Norgau, Germany) The value was obtained as an average of five measurements made at three points of the sample.

#### 2.2.4. Mechanical Analysis

Mechanical properties, including maximum strength and breaking strain, were ascertained using compression testing machine DVT GP UG 5 (Devotrans, Istanbul, Turkey). The stretching speed was 25 mm/min without preload pressure according to ASTM D5035-11. The working area of the samples was 10 × 40 mm.

Maximum strength, Fmax, N, was registered automatically. The average value was obtained from 5 measurements.

Elongation at break, *ε*, %, was calculated as:

(3) $$\varepsilon =\frac{∆l}{l_{0}}\times 100\%$$

where ∆*l*—the difference between the final and initial length of the sample; *l*_0_—the initial length of the sample. The average statistical error in measuring was ±0.2%.

#### 2.2.5. Differential Scanning Calorimetry (DSC)

Thermal characteristics including the melting enthalpy, melting temperature, degree of crystallinity were ascertained using the DSC 214 Polyma (Netzsch, Selb, Germany). The DSC temperature program included 2 heating cycles (from 20 °C to 220 °C) and 2 cooling cycles (from 220 °C to 20 °C). Samples were tested in an argon atmosphere, with a heating rate of 10 K/min and with a cooling rate of 10 K/min with sample weights of 6–7 mg.

Enthalpy of melting, *∆H*, J/g, was calculated by NETZSCH Proteus software according to the standard technique.

Temperature of melting, $T_{m}$, °C, was calculated by NETZSCH Proteus software according to the standard technique

Crystallinity degree, *χ*, %, was calculated as:

(4) $$\chi =\frac{∆H}{H_{PHB}}\times 100\%$$

where ∆*H*—melting enthalpy; *H_PHB_*—melting enthalpy of the initial ideal crystalline of the PHB, 146 J/g [33].

#### 2.2.6. Infrared Spectroscopy (FTIR)

The chemical composition of the materials was studied using a FTIR Lumos spectrometer (BRUKER, Berlin, Germany) at a temperature of 24 °C in the range of wavenumbers of 4000 ≤ ν ≤ 600 cm^−1^ in the mode of reflected light FTIR on the diamond crystal. The intensity of several absorption peaks related to polymer degradation (functional groups) was estimated by OPUS software (BRUKER, Berlin, Germany). The intensity of several absorption peaks related to polymer degradation (functional groups) was estimated. The resolution was 2 cm^−1^.

#### 2.2.7. Composting in Soil

The study of biodegradation in soil was carried out using reconstituted soil simulating real soil with a moisture capacity of 60%, at temperature 22 ± 3 °C, and a soil pH of 6. The soil was prepared by the standard technique [34,35].

The rate of the mass lost during biodegradation in soil, *R*, %, was calculated as:

(5) $$R=\frac{m_{i}-m_{s}}{m_{i}}\times 100\%$$

where $m_{i}$—mass of the initial sample; $m_{s}$—mass of the sample after the soil.

## 3. Results

**Figure 4:**
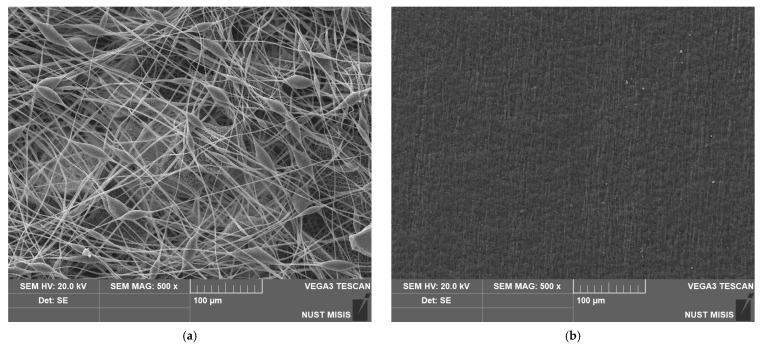
SEM images of the materials based on PHB obtained by different methods: (**a**) electrospinning, (**b**) pressing.

The method of production has a high impact on the appearance and structure of the surface of the material. Figure 4 shows a significant difference between the surface structures of nonwoven and pressed materials. It can clearly be seen that the film based on PHB has its own specific relief and low pores, but it cannot be comparable with the proportion of open pores in the inter-fiber space formed as a result of electrospinning. 

For quantitative comparison of the morphology of the surface of the two materials, the following indicators were used: surface density and porosity (Table 2). It should be mentioned that electrospun material and film of the same thickness 130 µm displayed a great difference in the friability of the materials’ volume and surface.

The degree of development of the surface certainly played an important role in the rate of biodegradation, given that all the conditions of the process were the same. So the first changes for nonwovens were recorded as early as on the 3rd day of composting, while for films noticeable changes were only recorded on the 30th day. Such observations indicate an increase in the rate of biodegradation depending on surface structure.

**Figure 5:**
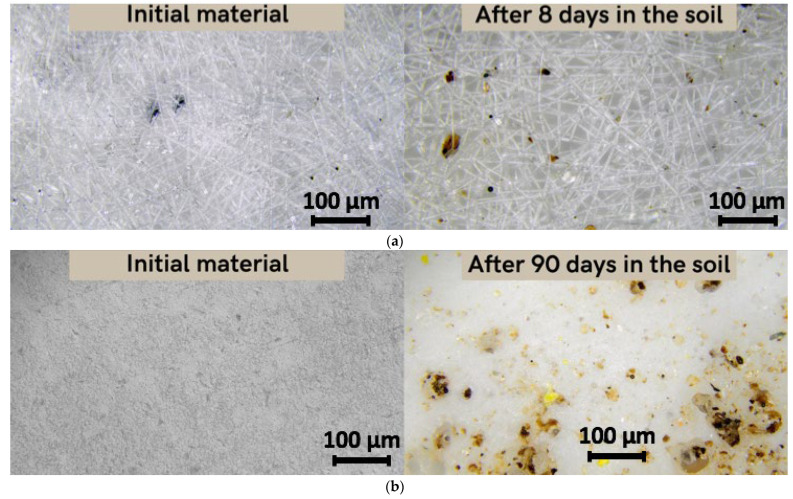
Microscopy of the original material and the material after placement in the soil (magnification 200): (**a**) fiber, (**b**) film.

As previously reported, it should be noted that there are three stages of PHB degradation in soil: transition from a high-molecular compound to monomers and oligomers; transition from monomers and oligomers to biomass; transition from biomass to CO_2_ and H_2_O [17]. Figure 5 shows the changes in the material’s structure at the first stage of degradation.

This stage is mainly represented by the mechanical action of soil adhesion and the activation of microbiota, and the beginning of oxidative and hydrolytic action under the influence of enzymes and soil components [27]. After 8 days of exposure to laboratory soil, the microstructure of the fibers changed significantly (Figure 5a). These changes were quantified by the morphological characteristics of the material. The average diameter of the fibers decreased from 5.6 microns to 5 microns and surface density decreased from 0.098 g/m^3^ to 0.047 g/m^3^. Moreover, inclusion of soil particles in the material’s structure and in pores was observed. However, for the film, the exposure time of 8 days had no effect on either the structure or the properties. In 90 days, however, the film had undergone noticeable changes. Surface density increased from 0.155 g/m^3^ to 0.192 g/m^3^. This increase could be explained by the fact that the soil particles are added to the material’s surface and the film could accumulate excess moisture or microbiotic component, something which is typical for the first stage of degradation in soil.

**Figure 6:**
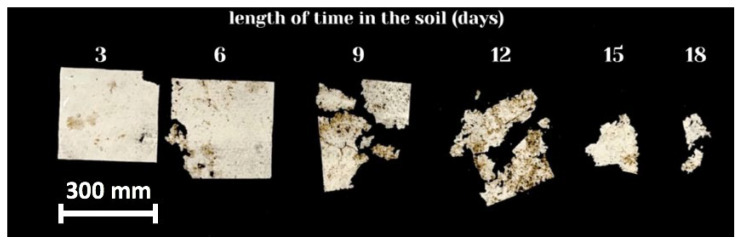
Biodegradation of fibrous material from PHB.

Figure 6 shows the duration of the biodegradation of the highly porous electrospun material based on PHB.

**Figure 7:**
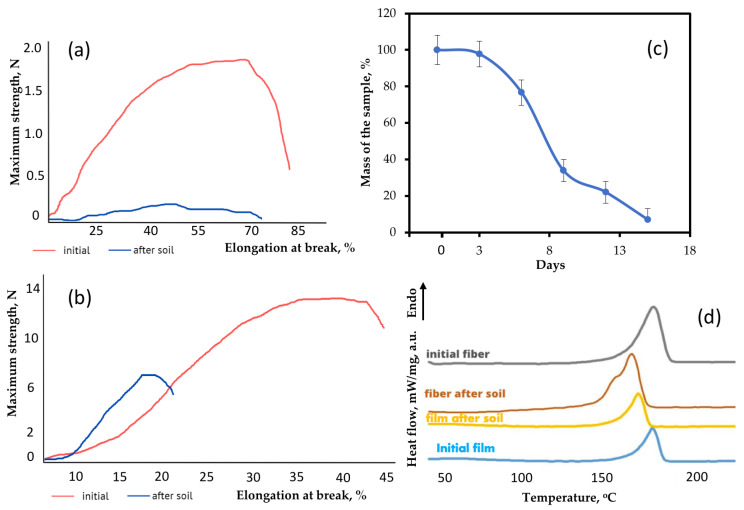
Changes in the structure and properties of materials: (**a**) stress–strain curves of fibrous material before and after the soil; (**b**) stress–strain curves of film before and after the soil; (**c**) the rate of the mass of the fibrous material based on PHB lost during biodegradation in the soil; (**d**) DSC curves of the material before and after the soil.

It should be noted that significant fragmentation and embrittlement of the sample were observed. In this process, it is difficult to assess the degree of mass loss in a nonwoven material sample. However, for the sake of the clarity of the biodegradation process, Figure 7c shows an averaged curve of the mass loss for three experiments. All electrospun materials based on PHB were fully disintegrated under composting conditions in less than 20 days.

Thus, not only was surface hydrolytic erosion observed [19], but disintegration of nonwoven samples with high porosity due to the space between the fibers (Figure 6) was also seen. Thus, there is a fragmentation stage corresponding to the beginning of the biodegradation process, which includes, at the first stage, the formation of monomers and oligomers as a result of chemical and mechanical actions; at the second stage, microbiological transformation to biomass; and at the last stage, chemical transformation to CO_2_ and H_2_O [18].

To establish the difference in the rates of biodegradation of film and electrospun fibrous material, various characteristics of the material were evaluated.

Mechanical properties are an important indicator of structural changes in the material. Changes in mechanical properties could also be triggered by the activity of microorganisms and by the influence of water and other factors. The effect exposure to the soil had on the mechanical properties of the polymer material are shown in Table 3.

It should be noted that nonwoven fabric and film with the same thicknesses have fundamentally different physical and mechanical properties. Materials based on PHB are generally considered to be quite fragile [36]. The features of the crystallization process often negatively affect the strength of the material, which is significantly inferior to synthetic polyesters [36]. The maximum strength of thin films based on PHB was 12.34 N. The strength of electrospun samples with the same thickness was decreased by a factor of nearly six. The main reason for this decrease is the fragility of individual fibers, which are easily torn, concentrating the residual stress of the undocrystallized areas when plasticizing additives are removed or in the case of post-treatment [37]. However, the decrease in strength is compensated for by an increase in maximum elongation. Table 3 shows that elongation at break of fibrous material is higher by almost 50%. Despite the fact that individual fibers tear more easily than a monolithic film, fibers provide higher flexibility in a whole system of nonwoven material [38]. Fibers have a certain degree of freedom, the restriction of which depends on gluing, thickening and other morphological defects [39]. In general, nonwoven fabric based on PHB is able to withstand larger elongations than a film of the same thickness due to the structural organization of the fibrous layer, as can be seen from the results.

Particular attention should be paid to the contribution of composting to the change in mechanical properties. Figure 7a shows changes in the stress–strain curves of the electrospun material after 8 days of exposure and Figure 7b shows changes the in stress–strain curves films after 90 days of exposure. These time periods were chosen due to the need to note changes in the first stage of biodegradation.

After exposure to the soil, the strength of the fibrous material decreased by 90% and the strength of the film decreased by 49%. The elongation at break also decreased by 26% for the fiber and by 60% for the film. The monolithic structure of the film was found to be to be more resistant to exposure and its biodegradation is slower. However, a significant embrittlement of the film, which was confirmed by a more intense decrease in elongation, indicates a chemical process occurring in the material. Although biodegradation in film samples proceeds according to the mechanism of surface destruction (Figure 2), the hydrolytic process could appear in whole system through the pores. The proportion of open pores in nonwoven fibrous material is many times higher than that in film, and the mechanism is approaching bulk degradation (Figure 2).

These data indicate a significant decrease in the strength and strain of polymer materials [40]. It is clearly seen that the method of polymer production makes a significant contribution to the rate of biodegradation. It should be mentioned that the key role in this process may be played by the surface area and the degree of accessibility of the polymer to soil microorganisms and chemical substances. The surface area of the fibrous material is many times larger than the surface area of the film.

Confirmation of this proposal follows from the analysis of the supramolecular structure using the DSC method. This method makes it possible to determine and investigate melting and crystallization temperatures [41]. Figure 7d shows the DSC curves of the material after biodegradation in soil. It is important to note that the method by which the PHB is obtained makes a significant contribution to the crystallization process and the perfection of the crystal structure of PHB [36]. It can clearly be seen that the shape of the melting peaks of the film and the fibrous material is quite similar, but the degree of crystallinity of the fibers is slightly higher. The crystallinity degree for the film and the fibrous material was 56.3% and 58.5%, respectively. The melting temperature of the samples was 174.4 °C and 174.5 °C, respectively.

**Figure 8:**
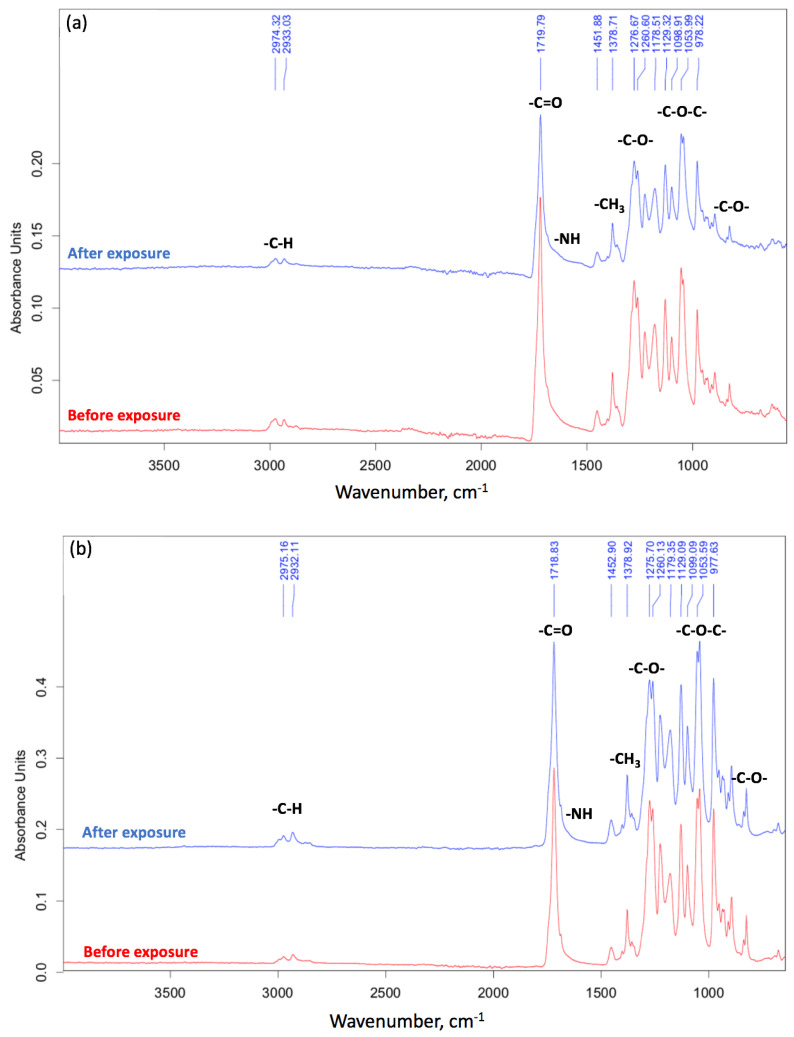
The FTIR spectra: (**a**) fiber, (**b**) film.

It was found that the crystallinity degree of the film decreased by almost a factor of two from 58.5% to 28% during exposure to the soil, while the crystallinity degree of the fibrous material decreased from 56.3% to 45%. A decrease in crystallinity is an important marker of the destruction of the crystalline phase of the polymer [9]. As it can be seen from Figure 8, the shape of the melting peak of the fibrous material changed a great deal. Despite a slight decrease in the degree of crystallinity of the fibers, it can clearly be seen that separation of the crystalline fraction occurred. After exposure to the soil, the crystallites began to melt at a lower temperature and some of them formed a low-temperature shoulder in the range of 140–160 °C. This indicates the availability of the crystalline phase for the effects of biodegradation factors. The melting point of the fibrous material decreased from 174.5 °C to 168.5 °C. which confirms the fragmentation of crystallites in the process of biodegradation. Smaller and defective crystalline fractions melt at lower temperatures [36]. At the same time, the melting point of the film decreased from 174.4 °C to 170.6 °C. Thus, a decrease in the melting point indicates a restructuring of the crystalline phase. Additionally, the decrease in the proportion of the crystal fraction was more noticeable. The results of the DSC are consistent with changes in physical and mechanical properties, since the structure and perfection of the crystalline phase largely affect the elongation and strength of the whole material. This indicates a noticeable and rapid effect from the soil microbiota, which is able to attack both the amorphous and crystalline phases more actively [42].

In addition, the FTIR method was able to confirm biodegradation [4]. The FTIR spectra are shown on Figure 8.

The general decrease in the intensity of the peaks after biodegradation is primarily due to the warping of the surface and the change in its relief under the action of the soil microbiota [43]. The most pronounced changes for the peaks at 1721 cm^−1^ (C=O group) and 1052 cm^−1^ (C-O-C group) for PHB at the first stages of biodegradation should be noted. Finally, a decrease in intensity of 1278 cm^−1^ (CH_3_ group) is expected for a more chemically stable part of the PHB chain [36]. In other works, the carboxyl index is used to characterize biodegradation, represented by the ratio of intensities of 1721 cm^−1^ and 1278 cm^−1^ [44]. The carbonyl index of PHB generally remains stable during the first stage of degradation (about 20 days) [45]. So, for nonwoven fabric it was 1.15 before degradation and 1.10 after 8 days of degradation (Figure 8a). Two processes may take place on the surface of the PHB in soil: accumulation on the surface of carbonyl groups resulting from the ester linkage hydrolysis [45] and the overall decrease of C=O groups as a consequence of their transition to -OH groups. These processes probably compensate for each other. At the same time, the peak of the ester linkage (1052 cm^−1^) has changed its shape and is difficult to identify. These observations indicate a break in the PHB chain and chemical changes that are usual for enzymatic hydrolysis [18].

For the film, the carbonyl index was 1.73 before degradation and 1.44 after 90 days of degradation (Figure 8b). These observations are already consistent with ideas about the process of biodegradation. This is consistent with the fact that after the first stage of biodegradation (about 20 days), the oligomers obtained as a result of the enzymatic hydrolysis of ester bonds leave the surface of the film [45]. Moreover, in the region of 900–700 and 1700–1500 cm^−1^, noticeable changes are observed for fibrous material (Figure 8a). This fact suggests the breakage of C-C bonds and the accumulation of OH groups, processes which are able to proceed faster when there is a greater availability of PHB macromolecules in the entire volume of the sample. This also confirms our ideas about the mechanism of biodegradation, something which proceeds in accordance with bulk degradation time.

Therefore, it is possible to generically characterize the biodegradation of nonwoven electrospun material as bulk degradation which occurs in the whole volume of the sample, while the degradation of the film proceeds according to a well-studied mechanism of surface degradation. In both cases, degradation is initiated from the polymer surface, having a significant effect on the amorphous and crystalline phases of the semi-crystalline PHB. But for a fibrous material, the contribution of the surface is so great that it accelerates degradation several times, leading to greater changes in the supramolecular and chemical structures than in the case of a film.

This theory is confirmed by the results obtained and by the differences in the degree of crystallinity. The crystallinity degree of the film decreased from 58.5% to 28% during 90 days in the soil, and the material was still comprehensive but fragile. At the same time, the crystallinity degree of the fibrous material decreased from 56.3% to 45% during 8 days, but the material had already disintegrated into small fragments. Such dynamics fully correspond to inversely proportional changes in mechanical properties. The elongation at break of the film decreased from 45.8% to 19.6% during 90 days in the soil. At the same time, the elongation at break of the fibrous material decreased from 67.5% to 49.9% during 8 days. At the same time, the high fragmentation of the nonwoven sample after 8 days in the soil is caused by a catastrophic decrease in strength to 0.16 N. The strength of the film was more than 6 N, which is almost three times higher than that of the fibrous material before the start of testing in the soil, with the samples of the same thickness.

## 4. Conclusions

Poly-3-hydroxybutyrate is a perfectly biocompatible polyester which does not cause environmental damage since its biodegradation products are water and CO_2_ [46,47]. Despite its low mechanical properties and low elasticity, in comparison with large-tonnage polymers, and taking into account its biodegradation abilities, it could be used in biomedicine, food eco-packaging, etc. [48].

This work established consistent patterns of changes in the supramolecular structure of the PHB, the chemical structure of the surface, and the physical and mechanical properties of the material during biodegradation in soil. Particular attention was paid to the contribution of the surface structure to the rate of biodegradation.

It was shown that a high degree of surface development, as in a nonwoven fibrous material, with a porosity of more than 80% and a high proportion of open pores, is able to disintegrate in the soil several times faster than a monolithic film material with a pore fraction of less than 10% with samples of the same thickness.

Thus, it is necessary to note the high contribution of the surface to the rate of biodegradation of the material. This leads to prospects for the creation of new materials with a wide range of applications and with controlled stability and speed of biodegradation.

## Figures and Tables

**Table 1:** Various additives.

Additives	Effect
Glycerol-based additives: glycerol triacetate (GTA), glycerol tributyrate (GTB) [10]	Act as nucleating agents, thermal stability
Hydroxyvalerate and hydroxyhexanoate [11]	Increased toughness
Triethyl citrate [12]	Improves mechanical, dynamic-mechanical and thermal properties
Acetyl o-tributyl citrate (ATBC) and tributyl citrate (TBC) [13]	Improvement of thermal, mechanical and barrier properties
Sugarcane bagasse [14]	cheaper materials, good fertilizers

**Table 2:** Morphology of the materials based on PHB.

Material	Surface Density, (±SD, n=10) δ, g/m^3^	Porosity, (±SD, *n* = 10) W,%
Fibrous material	0.098 ± 0.01	80 ± 2.0
Film	0.155 ± 0.03	6 ± 1.0

**Table 3:** Mechanical properties of the materials.

Material	Maximum Strength, Fmax, N	Elongation at Break, ε, %
Fibrous material	1.91	67.53
Fibrous material after soil (8 days)	0.16	49.93
Initial film	12.34	45.75
Film after soil (90 days)	6.31	19.61

## Data Availability

Not applicable.
